# The clinical differences between traumatic and NOS chronic subdural hematoma

**DOI:** 10.3389/fneur.2024.1453629

**Published:** 2024-10-17

**Authors:** Yongxiang Yang, Xiansong Zhu, Tao Yang, Kexia Fan, Jingmin Cheng, Yuan Ma

**Affiliations:** ^1^Department of Neurosurgery, The General Hospital of Western Theater Command, Chengdu, China; ^2^Institute of Biomedical Engineering, College of Medicine, Southwest Jiaotong University, Chengdu, China

**Keywords:** chronic subdural hematoma, trauma, clinical characteristics, clinical outcome, burr-hole craniotomy

## Abstract

**Objective:**

Chronic subdural hematoma (CSDH) is a common neurologic disorder with increasing incidence, which can be preceded by head trauma or occur in the absence of trauma. In order to deeply understand the clinical characteristics of this disease, we conducted this retrospective study to explore the clinical differences between traumatic and not otherwise specified (NOS) CSDH.

**Methods:**

According to the inclusion and exclusion criteria, 168 traumatic CSDH patients and 133 NOS CSDH patients were recruited from January 2015 to October 2023 in our cohort. The collected data and compared parameters including baseline clinical features and radiological outcomes of hematoma within 24 h of hospital admission, as well as the treatment method and clinical outcome of traumatic and NOS CSDH patients.

**Results:**

Compared to NOS CSDH patients, the average age was younger, epilepsy was more frequent, asymptomatic cases were more common, and the taking of anticoagulants and antiplatelet drugs were rarer in traumatic CSDH patients (all *P* < 0.05). However, no differences were found in the radiological presentations of hematoma at admission, the treatment methods and clinical outcomes of traumatic and NOS CSDH patients (all *P* > 0.05).

**Conclusion:**

Traumatic CSDH patients were more likely to be asymptomatic or have seizures, while NOS CSDH were more common in elder people and in individuals with the history of taking anticoagulants and antiplatelet drugs. The treatment methods and clinical outcomes were similar in traumatic and NOS CSDH patients.

## 1 Introduction

Chronic subdural hematoma (CSDH) is a common neurologic disease, which is characterized by the pathological collection of blood and blood breakdown products in the subdural space (1, 2). CSDH is often induced by head trauma via tearing the bridging veins with subsequent bleeding and hematoma creating, but it can also occur without trauma history in about 30%−50% cases (3–5). Apart from trauma, other risk factors that might be related to the onset of CSDH including the use of anticoagulants and antiplatelet drugs, alcohol abuse, cerebrospinal fluid shunts and so on (6, 7). CSDH patients with different inciting events might have various clinical characteristics. Until now, it is unclear whether there are differences in clinical features and treatment outcomes between traumatic and not otherwise specified (NOS) CSDH patients. Hence, it is of great potential significance to explore the clinical differences between traumatic and NOS CSDH.

CSDH patients can present with variable clinical manifestations including headache, nausea or vomiting, limb weakness, sensory disturbance, seizure, cognitive impairment, and so on (8–11). These symptoms result from the accumulation of blood in subdural space over time, the ongoing processes such as angiogenesis, fibrinolysis and inflammation subsequently, the elevation of intracranial pressure and the compression of brain parenchyma after 4–7 weeks ultimately (9–12). The diagnosis of CSDH is based on the combination of above clinical symptoms and radiological investigation, mostly computed tomography (CT) scan. CT not only reflects the shape, density and volume of the hematoma, but also reveals the natural development of CSDH from a homogeneous type into a laminar, then separated type, and finally be absorbed as a trabecular hematoma (13, 14). The treatment strategy of CSDH consists of surgical evacuation through burr-hole craniotomy, the medication therapy including dexamethasone and atorvastatin, and conservative observation. Surgical treatment is appropriate for patients with impeding, progressive, or severe neurological symptoms, which can obtain a fast neurological improvement via an immediate relief of pressure on the ipsilateral hemisphere (14, 15). Medication therapy is used as an alternative therapy for symptomatic patients, in an attempt to avoid surgery or reduce postoperative recurrence risk (14). Observational therapy is applied to treat asymptomatic patients or patients with mild symptoms. Based on the above mentioned literature, we think it is meaningful to analyze the differences of clinical symptoms, CT results, treatment strategies, and treatment outcomes between traumatic and NOS CSDH patients.

To our knowledge, few studies have identified the clinical differences between traumatic and NOS CSDH. Herein, the present study was conducted to compare parameters including baseline clinical features and radiological outcomes of hematoma, as well as the treatment method and clinical outcome of traumatic and NOS CSDH patients. The aim of this study is to clarify the clinical differences between traumatic and NOS CSDH, and further to provide a theoretical basis for earning more effective treatments and better outcomes.

## 2 Materials and methods

### 2.1 Setting

We conducted this retrospective study and collected the clinical data of traumatic and NOS CSDH patients at the General Hospital of Western Theater Command in Chengdu, China. Traumatic CSDH refers to the main symptoms that lead to the hospitalization of patients are closely related to head trauma such as fallen, traffic accidents during the past days or weeks. The main symptoms include persistent headache or dizzy, transient disturbance of consciousness, reduced recent memory, limb weakness, seizure, cognitive impairment, and so on. NOS CSDH refers to patients present as above symptoms, but without the head trauma. The department of neurosurgery in the hospital have professional neurosurgeons and nurses, who are in charge of formulating treatment and care plans for CSDH patients. Ethics committee of the faculty of The General Hospital of Western Theater Command gave permission for this research. All the studying processes were carried out in accordance with the approved guidelines.

### 2.2 Patients

Traumatic and NOS CSDH patients were recruited from the inpatient service of The General Hospital of Western Theater Command in Chengdu, China. The collected data including complete admission and hospitalization records of all patients with CSDH from January 2015 to October 2023. The inclusion criteria including: (1) Age ≥ 18 years. (2) The discharge diagnosis was CSDH. (3) Patients can clearly recall and state the course of disease, especially the course of head trauma event of traumatic CSDH. Patients with following situations were excluded: (1) The admission and hospitalization information was incomplete. (2) Patients cannot recall the course of disease, especially cannot clarify the relation between the onset of CSDH and head trauma. (3) Presence of extracranial injury (such as orthopedic/cardiac/chest/abdominal/pelvis traumatic injury and so on). (4) Pre-existing severe cardiac diseases (such as myocardial ischemia/infarction, heart failure). (5) Combined with liver/renal/lung failure, hematological disease, infection disease, malignancy, and pregnancy.

### 2.3 Clinical care of patients with CSDH

Once patients arrived at the department of neurosurgery in our hospital, standard treatments and management were carried out immediately. All the CSDH patients received comprehensive neurological evaluation and underwent cranial CT scan subsequently. And, repeat CT scan was conducted when patients showed the indication of clinical deterioration or the sign of intracranial pressure elevation. Moreover, other routine clinical examinations including chest X-Ray, abdomen ultrasound, electrocardiogram and laboratory tests including hematology, urine and feces analysis, lipid, and coagulation profile, multiorgan (cardiac, liver, and renal) function analysis were conducted within 12 h after patients were hospitalized. Once the patient was diagnosed as CSDH, appropriate treatment including surgical hematoma evacuation through burr-hole craniotomy, the medication therapy including dexamethasone/atorvastatin, and conservative observation would be conducted.

### 2.4 Data collection

All parameters that might be different between traumatic and NOS CSDH according to our existing knowledge and previous literature were analyzed in this study. The collected data including baseline clinical features and radiological outcomes of hematoma within 24 h of hospital admission, as well as the treatment method and clinical outcome of traumatic and NOS CSDH patients. Baseline clinical features involved demography, main symptoms, initial GCS, mode of trauma and medical history. Radiological outcomes of hematoma included hematoma location, maximum thickness, midline shift, and hematoma volume based on the CT presentation. Treatment methods consisted of borehole drainage, atorvastatin and dexamethasone. And, clinical outcomes were evaluated by neurological function, hospital stays, and total costs.

### 2.5 Data analysis

Measurement data was expressed as mean values ± standard deviations (M ± SD). Differences between two groups were analyzed by Unpaired *t* test with Welch's correction. Enumeration data was analyzed by Chi-squared test. SPSS version 18.0 software (SPSS Inc., USA) was used to perform the analysis, and two-tailed *P* < 0.05 was considered statistically significant. Photoshop software (Adobe Software, Inc., USA) was used to draw the figure.

## 3 Results

### 3.1 Patients selection

**Figure 1:**
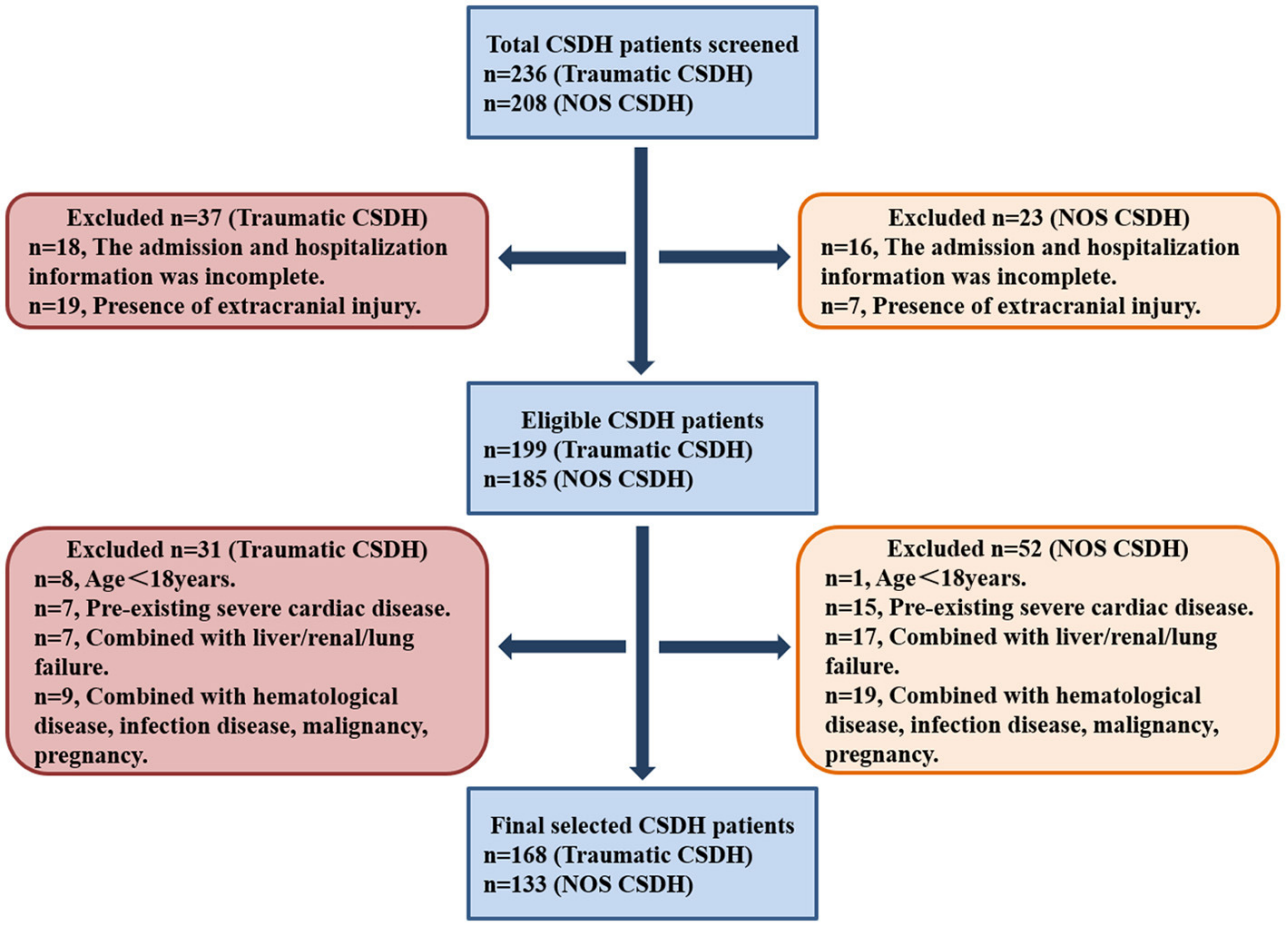
The selection flowchart of traumatic and NOS CSDH patients. NOS, not otherwise specified.

All CSDH patients were screened from the inpatient service of The General Hospital of Western Theater Command in Chengdu from January 2015 to October 2023 by using ICD-9 procedural code terminology. CSDH patients were further selected according to the inclusion and exclusion criteria as described in “Materials and Methods” section. At last, 168 traumatic CSDH and 133 NOS CSDH patients were recruited. The selection flowchart was illustrated in Figure 1.

### 3.2 Traumatic and NOS CSDH patients had different baseline clinical features

Traumatic and NOS CSDH patients had similar gender distribution, but the mean age of the latter was older (65.9 ± 16.3 y vs. 70.8 ± 13.0 y, *P* = 0.005). Traumatic and NOS CSDH patients had similar mean GCS score and severity of illness, but had different main symptoms. Results indicated that epilepsy was more frequent and asymptomatic cases were more common in traumatic CSDH patients compared to NOS CSDH patients (7.7% vs. 3.0% and 15.5% vs. 6.8%, *P* < 0.001). The most common mode of trauma in traumatic CSDH patients was “falls” (61.3%). As for the medical history, the taking of anticoagulants and antiplatelet drugs were more common in NOS CSDH patients than in traumatic CSDH patients (9.0% vs. 1.2%, *P* = 0.001; 13.5% vs. 4.2%, *P* = 0.003). Detailed data was showed in Table 1.

**Table 1:** Baseline characteristics of traumatic/NOS CSDH patients at admission.

**Variable**	**Traumatic CSDH**	**NOS CSDH**	**T/χ^2^**	***P-*value**
Gender (*n*, %)			4.639	0.031
Male	147 (87.5%)	104 (78.2%)		
Female	21 (12.5%)	29 (21.8%)		
Age (y)	65.9 ± 16.3	70.8 ± 13.0	−2.803	**0.005** ^*^
Main symptoms (*n*, %)			23.613	**<0.001** ^*^
Headache & dizziness	74 (44.1%)	77 (57.9%)		
Limb weakness	55 (32.7%)	43 (32.3%)		
Epilepsy	13 (7.7%)	4 (3.0%)		
No symptoms	26 (15.5%)	9 (6.8%)		
Initial GCS	14.1 ± 1.7	14.2 ± 1.9	−0.346	0.715
Mode of trauma			/	**/**
Traffic accidents	65 (38.7%)	/		
Falls	103 (61.3%)	/		
Medical History (*n*, %)				
Hypertension	57 (33.9%)	51 (38.3%)	0.630	0.428
Coronary heart disease	11 (6.5%)	6 (4.5%)	0.578	0.447
Diabetes	25 (14.9%)	13 (9.8%)	1.755	0.185
Anticoagulant drug	2 (1.2%)	12 (9.0%)	10.268	**0.001** ^*^
Antiplatelet drug	7 (4.2%)	18 (13.5%)	8.553	**0.003** ^*^
Smoking history	87 (51.8%)	54 (40.6%)	3.729	0.053
Drinking history	88 (52.4%)	62 (46.6%)	0.987	0.321

CSDH, chronic subdural hematoma; NOS, not otherwise specified; GCS, Glasgow Coma Scale. *P* < 0.05 represents statistically significant (marked with ^*^ and bold).

### 3.3 Traumatic and NOS CSDH patients had similar radiological presentations

As shown in Table 2, there were no significant differences in the CT presentations of hematoma between traumatic and NOS CSDH patients at admission. The most common hematoma location of traumatic and NOS CSDH patients was left side (42.3% vs. 48.1%), followed by right side (35.1% vs. 35.3%), and bilateral (22.6% vs. 16.6%). In traumatic and NOS CSDH patients, the mean value of maximum thickness of hematoma was 18.7 ± 8.2 mm and 20.0 ± 7.5 mm, midline shift of brain was 9.3 ± 4.2 mm and 9.8 ± 4.7 mm, and hematoma volume was 102 ± 64 ml and 109 ± 71 ml. All in all, above data showed that traumatic and NOS CSDH patients had similar CT presentations of hematoma.

**Table 2:** Radiological parameters of traumatic/NOS CSDH patients at admission.

**Variable**	**Traumatic CSDH**	**NOS CSDH**	**T/χ^2^**	***P-*value**
Hematoma location (*n*, %)			1.945	0.378
Left side	71 (42.3%)	64 (48.1%)		
Right side	59 (35.1%)	47 (35.3%)		
Bilateral	38 (22.6%)	22 (16.6%)		
Maximum thickness (mm)	18.7 ± 8.2	20.0 ± 7.5	−0.881	0.381
Midline shift (mm)	9.3 ± 4.2	9.8 ± 4.7	−0.636	0.514
Hematoma volume (ml)	102 ± 64	109 ± 71	−0.742	0.453

CSDH, chronic subdural hematoma; NOS, not otherwise specified.

### 3.4 Traumatic and NOS CSDH patients had similar treatment and clinical outcome

As shown in Tables 3, 4, the rate of borehole drainage in traumatic and NOS CSDH patients was similar (78.6% vs. 73.7%, *P* = 0.321). And, the rate of medication therapy including atorvastatin and dexamethasone in two groups was similar as well (62.5% vs. 67.7%, *P* = 0.351; 16.7% vs. 16.5%, *P* = 0.977). The outcome of neurological function at discharge was also similar in traumatic and NOS CSDH patients (*P* = 0.909). Furthermore, the mean hospital stays and total costs of traumatic and NOS CSDH patients were almost equal (9.9 ± 5.4 days vs. 10.1 ± 6.6 days, *P* = 0.831; 2.7 ± 2.4 wan yuan vs. 2.6 ± 2.6 wan yuan, *P* = 0.914). These findings indicated that traumatic and NOS CSDH patients had similar treatment and clinical outcomes.

**Table 3:** Treatment of traumatic and NOS CSDH patients.

**Variable**	**Traumatic CSDH**	**NOS CSDH**	**T/χ^2^**	***P***
**Borehole drainage (** ***n*** **, %)**			0.984	0.321
With	132 (78.6%)	98 (73.7%)		
Without	36 (21.4%)	35 (26.3%)		
**Atorvastatin (** ***n*** **, %)**			0.869	0.351
With	105 (62.5%)	90 (67.7%)		
Without	63 (37.5%)	43 (32.3%)		
**Dexamethasone (** ***n*** **, %)**			0.001	0.977
With	28 (16.7%)	22 (16.5%)		
Without	140 (83.3%)	111 (83.5%)		

CSDH, chronic subdural hematoma; NOS, not otherwise specified.

**Table 4:** Clinical outcome of traumatic and NOS CSDH patients at discharge.

**Variable**	**Traumatic CSDH**	**NOS CSDH**	**T/χ^2^**	***P***
Neurological function (*n*, %)			0.19	0.909
Improvement	103 (61.3%)	84 (63.2%)		
No change	48 (28.6%)	35 (26.3%)		
Deterioration	17 (10.1%)	14 (10.5%)		
Hospital stays (days)	9.9 ± 5.4	10.1 ± 6.6	−0.213	0.831
Total costs (wan yuan)	2.7 ± 2.4	2.6 ± 2.6	0.109	0.914

CSDH, chronic subdural hematoma; NOS, not otherwise specified.

## 4 Discussion

This retrospective study explored the clinical differences between traumatic and NOS CSDH patients. Major results were as follows: (1) Compared to NOS CSDH patients, the average age was younger, epilepsy was more frequent, asymptomatic cases were more common, and the application of anticoagulants and antiplatelet drugs were rarer in traumatic CSDH patients. (2) No differences were found in the radiological presentations of hematoma at admission, the treatment methods and clinical outcomes of traumatic and NOS CSDH patients. These findings are meaningful for deeply understanding the clinical characteristics of CSDH.

CSDH is a common neurosurgical disease, with an incidence of 17.2–20.6 per 100,000 persons per year, which is higher in the elderly and likely to reach 17.4 per 100,000 individuals by 2030 due to aging population and increasing use of anticoagulants (9, 16, 17). CSDH is conventionally considered to be a part of traumatic brain injury, which can be induced by isolated head trauma or develop following a traumatic subdural hygroma or mild acute subdural hematoma (18). In our study, traumatic CSDH was defined as patients presented with the main symptoms such as persistent headache, transient disturbance of consciousness or limb weakness that were closely related to head trauma events such as fallen, traffic accidents during the past days or weeks. In the clinical practice, we found a significant proportion of CSDH patients were indeed undergoing head trauma before the development of clinical symptoms of CSDH. Usually, the head trauma is more frequent in younger CSDH patients than in elderly CSDH patients (19). The most common event of trauma in elderly CSDH patients is fallen, but in younger ones is traffic accident (20, 21). The higher rate of traumatic CSDH in younger ones might be explained by the fact that most of younger individuals are in a state of working, movement and exercising, resulting in a higher rate of head trauma caused by unintentional injury, traffic accident and fallen. Consistent with these literatures, our study indicated that the mean age of traumatic CSDH patients was younger than NOS CSDH patients, and the most common mode of trauma was “falls.” The pathological mechanism in the association of elderly, trauma and CSDH might be an increase in extracerebral volume after age increasing and brain weight declines, which leads to an easily tear of bridging veins and subsequent hematoma formation after trauma. Another cause of the elevated incidence of CSDH in elderly individuals is the increasingly use of anticoagulants and antiplatelet drugs. Many studies reported that a proportion of CSDH patients, especially elderly ones had the prior treatment with anticoagulant and antiplatelet drug (6, 22–24). The rate of CSDH patients treated with anticoagulant drugs was 2.5%−40.6%, and with antiplatelet drugs was 4.5%−44%, which was higher in the elderly and different in different countries (19). We found the rate of taking anticoagulant and antiplatelet drugs in NOS CSDH patients was 9.0% and 13.5% respectively, which was obviously higher than in traumatic CSDH patients. Taking together, these results indicated that NOS CSDH patients were more common in the elderly, and had a higher rate of taking anticoagulant/antiplatelet drugs, compared to traumatic CSDH patients.

CSDH patients often begin to manifest symptoms after the intracranial volume is overwhelmed by the expanding hematoma, which is usually diverse and includes headache, gait disturbance, hemiparesis, and cognitive problems (25, 26). One latest study indicated that the most prevalent symptoms in CSDH patients were focal neurological deficit (46%), headache (41%), gait disorder (31%), and cognitive complaint (31%) (27). Similarly, our study found that headache and limb weakness were the most common symptoms in both traumatic and NOS CSDH patients. Moreover, our results indicated that epilepsy was more frequent and asymptomatic cases were more common in traumatic CSDH patients, compared to NOS CSDH patients. This result might be explained by the fact that traumatic CSDH is one of the major predictor of seizure (28). Traumatic CSDH is one type of traumatic brain injury, which is a well-established risk factor for seizure (29). The severity of brain trauma was not only a predictor for early seizure, but also a major predictor for late seizures and post-traumatic epilepsy (28). Accordingly, epilepsy was more frequent in traumatic CSDH patients might be explained the fact that those patients were often combined with a mild or moderate brain trauma, a potential inducement of late seizures and post-traumatic epilepsy. Seizure is a rare and prominent sign, which reflects the extreme clinical manifestation of CSDH. Some studies demonstrated that CSDH patients with seizure would have a worse outcome (28), but another study did not find an association between the seizure and poor outcome of CSDH (27). Moreover, seizures after CSDH surgery, mainly refer to burr hole craniotomy, were associated with postoperative complications, higher mortality and poorer clinical outcomes at follow-up (30). And, larger post-operative depressed brain volume was the only factor independently associated with suspected postoperative seizure, and it could help identify a subgroup of CSDH patients with higher susceptibility to epileptic events (31). Although seizure is an offensive complication after surgery in CSDH patients, no studies have demonstrated the postoperative use of antiepileptic drugs can lead to better clinical outcomes.

Non-contrasted head CT is the mainstay and initial diagnostic method for CSDH, which can reflect the radiological outcomes of hematoma included hematoma location, maximum thickness, midline shift and hematoma volume. Our study showed that traumatic and NOS CSDH patients had similar CT presentations of hematoma, and the most common hematoma was left side, followed by right side and bilateral. According to the hematoma density on CT scan, CSDH is categorized into five subtypes including high, moderate, low, mixed, and layering (32). According to the internal architecture of the hematoma on CT scan, CSDH is categorized into four subtypes including homogeneous, laminar, separated, and trabecular, which is considered to reflect the four stages of the natural progression of the disease (33). Head CT at the time of trauma preceding CSDH usually showed thin subdural effusion, which was often close to the detection limit of CT immediately, but became more apparent from the day after the injury (34). Moreover, some researchers found no benefit for routine follow-up CT after surgery for CSDH over CT performed only in patients with clinical deterioration or persisting neurologic deficits (35). Based on these literatures, conducting in-depth research on the preoperative CT classification and postoperative follow-up CT is essential for the diagnosis of CSDH and the prediction of recurrence after surgery.

The treatment strategy of CSDH patients consists of surgical evacuation of the hematoma, the medication therapy including dexamethasone and atorvastatin, and conservative observation. Surgical intervention is recommended for CSDH patients who manifest as neurologic symptoms with associated clinical or radiographic evidences of cerebral compression (36). The main surgical treatment in symptomatic patients including twist drill craniotomy (TDC), burr-hole craniotomy (BHC) and craniotomy, which can evacuate the hematoma effectively and is often combined with the placement of a subdural or sub-periosteal drain (15, 37, 38). Generally, the outcomes after surgery of symptomatic CSDH are favorable, with rapid clinical improvement occurring in over 80% of patients (39). However, the overall surgical mortality ranges widely from approximately 0% to 32%, and the operative morbidity ranges from approximately 3% to 12% (36, 40, 41). TDC is the most minimally invasive surgery for CSDH, which creates a small (<5 mm) burr hole and is usually performed at bedside through local anesthesia (25). The advantages of TDC include the minimum invasiveness, the avoidance of general anesthesia and the lower overall procedural risk, particularly in patients older than 60 years or have medical comorbidities (42, 43). The morbidity and mortality associated with TDC was 2.5%−4.4% and 2.9%−5.1% respectively (40, 44), significantly lower than other surgical methods. However, the hematoma evacuation rate of TDC was obviously lower than other surgical techniques, resulting in a higher recurrence rate of CSDH ranging from 28.1%−31.3% (40, 44). BHC is the most common surgery for CSDH, which is performed by drilling one or two 12–14 mm burr holes on the cerebral convexity 5–8 cm apart firstly, and evacuating the hematoma by suction and irrigation subsequently (15, 45). Normal saline is the most commonly used irrigation solution, artificial cerebrospinal fluid and the solution at body temperature are two new-type irrigation solutions, which were reported to reduce the recurrence rate of CSDH (46, 47). BHC is most commonly performed under general anesthesia, though local anesthesia is a feasible alternative one with lower complication rates (48, 49). The morbidity and mortality of BHC was 4%−9.3% and 2.5%−3.7% respectively, and the recurrence rate of CSDH after BHC was 10.5%−12.0%, obviously lower than the recurrence rate after TDC (39, 40, 50). Craniotomy is the most invasive and surgically effective, but less frequently performed surgery for CSDH, which creates a skull bone window at 3–5 cm in diameter and incises the dura to evacuate the hematoma and allow fluid to drain out of the subdural space (51). The advantages of craniotomy include this technique can evacuate the hematoma thoroughly, excise the hematoma membrane and coagulate the bleeding point precisely under a direct vision (52). The morbidity and mortality correlated with craniotomy was 4%−12% and 4.6%−12.2% respectively, significantly higher than TDC and BHC (39, 40, 44, 50), the recurrence rate after craniotomy was 11%−19.4%, higher than BHC and lower than TDC (40, 41). Other newly developed surgical techniques including the “Hollow screw system” is applied as a less invasive surgery for elderly patients with CSDH (53), the endoscope is used for recurrent or complicated hematoma cases, and middle meningeal artery (MMA) embolization is considered a promising treatment for patients with initial and/or recurrent CSDH (54). Hollow screw system is a modification of TDC, in which a hollow screw is threaded through a twist-drill hole and is connected to a closed drainage system, without the insertion of a catheter into subdural space (53). One latest meta-analysis indicated that TDC with hollow screws was not inferior or superior to BHC in efficacy, but was safer and minimally invasive, which reflected in a lower incidence of acute brain hemorrhage, overall complication and longer hospital stays (55). Neuroendoscopy can be used in both BHC and craniotomy, which provides enhanced visualization of the hematoma, the trabeculae, and septation (56, 57). The visualization of neuroendoscopy can further facilitate more complete hematoma evacuation, more precise hematoma membrane excision and meticulous microscopic hemostasis, resulting in low complication rates and reduced recurrence rates (22, 58). MMA embolization can inhibit hematoma expansion and recurrence by embolizing the subdural neomembrane capillaries through the catheter-based endovascular intervention technique (59, 60). Several single-cohort and multi-center studies have demonstrated that MMA embolization in conjunction with surgical evacuation can effectively reduce recurrence rates and is associated with low procedural complication rates (61–65). The conservative observational approach and medical treatment is appropriate for asymptomatic CSDH patients who have mild symptoms such as headache only, but have no sign of cerebral compression. Glucocorticoid therapy, mainly refers to dexamethasone, has been proposed as an alternative, non-operative treatment for CSDH, which has the potential to block inflammation in subdural space, thereby impeding hematoma persistence and growth (66). Although glucocorticoids were reported to be safe and effective as a therapy for CSDH, two recent randomized controlled trials have demonstrated that dexamethasone resulted in fewer favorable outcomes and more adverse events than placebo (66, 67). Another effective medical treatment is atorvastatin, which could lead to a reduction in CSDH volume without the requirement for surgery and showed no adverse effects (68–72). One double-blind, randomized, placebo-controlled phase II clinical trial found the atorvastatin at 20 mg daily to be safe, effective and cost effective for non-surgically treating patients (68). Another open-label, evaluator-blinded trial found that compared with atorvastatin alone, the combination of atorvastatin and low-dose dexamethasone accelerated hematoma reduction and neurological improvement in CSDH patients (69). Moreover, atorvastatin administration after surgery was reported to reduce hematoma volume and improve neurological function, but barely reduced the recurrence rate of hematoma (70). On the contrary, one latest study demonstrated that an administration of atorvastatin of 20 mg daily for 4 weeks may be helpful in reducing the recurrence rate of CSDH following BHC surgery without any serious adverse effects (71). Our research found the rate of BHC in traumatic and NOS CSDH patients was 78.6% vs. 73.7%, and, the rate of atorvastatin therapy was 62.5% vs. 67.7%, dexamethasone therapy was 16.7% vs. 16.5%. At last, most of the traumatic and NOS CSDH patients acquired neurological function improvement at discharge.

Based on the above research findings, several potential implications for clinical practice might be worth mentioning. Firstly, detailed medical history of head trauma and taking of anticoagulants/antiplatelet drugs should be inquired carefully in younger and older CSDH patients respectively. Secondly, more attention should be paid to the prevention, diagnosis and treatment of epilepsy in younger CSDH patients in the perioperative period, especially the ones with medical history of head trauma. Thirdly, the selection of treatment protocols for traumatic and NOS CSDH patients should be mainly made according to the outcome of CT/MRI scan.

There are some objective limitations in this study should be considered. First, this study is an observational and retrospective one without active intervention and long-term follow-up visits. Second, the number of patients is relatively small and the ill condition of recruited patients is mainly mild to moderate as the mean GCS in two groups was about 14. Finally, BHC was the only surgical treatment for CSDH patients in this study, making the comparison of curative effects between different surgical methods to be impossible. Therefore, it is very necessary to conduct prospective, large sample and in-depth clinical study to explore this issue in the future.

In conclusion, this retrospective study explored the clinical differences between traumatic and NOS CSDH patients. Compared to NOS CSDH patients, the average age was younger, epilepsy was more frequent, asymptomatic cases were more common, and the taking of anticoagulants and antiplatelet drugs were rarer in traumatic CSDH patients. However, no differences were found in the radiological presentations of hematoma at admission, the treatment methods and clinical outcomes of traumatic and NOS CSDH patients. These findings are meaningful for deeply understanding the clinical characteristics of CSDH.

## Data Availability

The raw data supporting the conclusions of this article will be made available by the authors, without undue reservation.
